# Genetic Polymorphisms of Glutathione S-Transferase P1 (*GSTP1*) and the Incidence of Anti-Tuberculosis Drug-Induced Hepatotoxicity

**DOI:** 10.1371/journal.pone.0157478

**Published:** 2016-06-09

**Authors:** Shouquan Wu, You-Juan Wang, Xiaoyan Tang, Yu Wang, Jingcan Wu, Guiyi Ji, Miaomiao Zhang, Guo Chen, Qianqian Liu, Andrew J. Sandford, Jian-Qing He

**Affiliations:** 1 Department of Respiratory and Critical Care Medicine, West China Hospital, Sichuan University, Chengdu, Sichuan, China; 2 Physical Examination Center, West China Hospital, Sichuan University, Chengdu, Sichuan, China; 3 Division of Geriatrics, Sichuan Provincial People's Hospital, Chengdu, Sichuan, China; 4 Department of Laboratory Medicine, West China Hospital, Sichuan University, Chengdu, Sichuan, China; 5 Centre for Heart Lung Innovation, University of British Columbia and St. Paul’s Hospital, Vancouver, BC, Canada; Food and Drug Administration, UNITED STATES

## Abstract

**Background:**

Anti-tuberculosis drug-induced hepatotoxicity (ATDH) is one of the most common adverse effects associated with tuberculosis (TB) therapy. Animal studies have demonstrated important roles of glutathione S-transferases in the prevention of chemical-induced hepatotoxicity. The aim of this study was to investigate the relationship between single nucleotide polymorphisms (SNPs) of glutathione S-transferase P1 (*GSTP1*) and ATDH in TB patients.

**Methods:**

We used two independent samples for this genetic association study. In the initial prospective study, 322 newly diagnosed TB patients were followed up for three months after initiating anti-TB therapy. In an independent retrospective study, 115 ATDH patients and 116 patients without ATDH were selected to verify the results of the prospective study. Tag-SNPs of *GSTP1* were genotyped either with the MassARRAY platform or the improved multiple ligase detection reaction (iMLDR) method. The associations between SNPs and ATDH were analyzed by logistic regression analysis adjusting for confounding factors.

**Results:**

Of the 322 patients recruited in the prospective cohort, 35 were excluded during the 3 months of follow-up, and 30 were diagnosed with ATDH and were considered as the ATDH group. The remaining 257 subjects without ATDH were considered as the non-ATDH group. After correction for potential confounding factors, significant differences were found for rs1695 (A>G) under an allelic model (OR = 3.876, 95%CI: 1.258011.905; P = 0.018). In the retrospective study, rs1695 allele A also had a higher risk of ATDH (OR = 2.10, 95%CI: 1.17–3.76; P = 0.012). We only found rs4147581AA genotype under a dominant model was related to ATDH in the prospective study (OR = 2.578, 95%CI: 1.076–6.173; P = 0.034).

**Conclusions:**

This is the first study to suggest that *GSTP1* genotyping can be an important tool for identifying patients who are susceptible to ATDH. This result should be verified in independent large sample studies and also in other ethnic populations.

## Introduction

Tuberculosis (TB) remains a major worldwide public health problem. Approximately one third of population of the world is infected by mycobacterium tuberculosis (MTB). TB ranks as the second leading cause of death from an infectious disease worldwide, and there were 9.0 million new TB patients and 1.5 million TB deaths in 2013 [1]. The first-line drugs isoniazid (H), rifampicin (R), pyrazinamide (Z), ethambutol (E) and streptomycin (S) are considered the most effective drugs for TB treatment [2]. The standard chemotherapy regimen recommends a combination of HRZE for two months, followed by HR for four months (2HRZE/4HR) [3]. Despite this effective therapeutic approach, multi-drug regimens can increase the risk of severe adverse drug reactions such as hepatotoxicity, allergic reactions, gastrointestinal disorders, etc. [4].

One of the most prevalent adverse drug reactions encountered in the course of TB treatment is anti-TB drug-induced hepatotoxicity (ATDH), also named anti-TB drug-induced liver injury, which can result in early withdrawal from treatment, decreasing the effectiveness of therapy. The pathogenesis of ATDH is still poorly understood [5] and a better understanding of this adverse drug reaction may lead to individualized treatment. Known risk factors for ATDH include female sex, older age, HIV infection, alcohol intake and hepatitis B or C virus infection [6–9]. In addition, genetic factors also play critical roles in individual’s susceptibility to developing ATDH [9].

The glutathione S-transferase (*GST*) genes code for a superfamily of enzymes that participate in phase-II drug metabolism. GSTs play critical roles in the biological detoxification processes of numerous drugs including anti-TB drugs [10, 11]. They reduce the risk of drug-induced hepatotoxicity not only by catalyzing the conjugation reactions of toxic intermediary metabolites but also by stimulating toxicant elimination. Several studies in animal models demonstrated the important role of GSTs in the prevention of chemical-induced hepatotoxicity [12, 13].

A previous study has shown an increased risk of ATDH in individuals carrying the *GSTM1* null genotype but not the *GSTT1* null genotype [14]. Another study recently investigated the role of *GSTP1* in ATDH [15]. In this study, no association was found between *GSTP1* Ile105Val (rs1695A/G) polymorphism and ATDH, but it revealed that hypermethylation of CpG islands of the *GSTP1* promoter was related to ATDH [15]. The aim of the current study was to investigate the relationship between tag-single nucleotide polymorphisms (tag-SNPs) and haplotypes of *GSTP1* and ATDH in TB patients in a prospective study and an independent retrospective study.

## Methods and Materials

### Study design and patients

This study was approved by the Institutional Review Board of the West China Hospital of Sichuan University. Our study consisted of two samples. The initial sample was a prospective study and the other independent sample was a retrospective study which replicated the results of the prospective study. All patients were required to sign an informed consent. In the prospective study, a total of 322 newly diagnosed TB patients were recruited into this study between August 2012 and January 2016 at the West China Hospital (Chengdu, China). All TB cases were diagnosed by specialized physicians based on clinical symptoms, chest x-ray examination, smear tests and/or culture and/or polymerase chain reaction (PCR) of sputum/body fluid/tissue for MTB and/or good response to anti-TB therapy.

The eligibility criteria were: 1) signed written consent and agreement to be monitored regularly during anti-TB therapy. 2) no history of using anti-TB drugs in the past two years. 3) laboratory tests of liver function within normal limits before using anti-TB drugs. 4) no comorbidity with HBV, HCV or HIV infection, or cancer or other diseases may affect liver function. Patients with following conditions were excluded from the final analysis: lost to follow-up during the first three months of anti-TB treatment; having diseases after enrolment that required the use of other hepatotoxic drugs simultaneously in the study period or that can influence liver function. The remaining patients were stratified into those who satisfied the criteria for ATDH and those who did not.

In the independent retrospective study, 115 cases and 116 controls groups were selected by the same inclusion and exclusion criteria from August 2012 and January 2016.

All patients were initially treated with standard anti-TB treatment protocol including isoniazid 300 mg/day, rifampicin 450 mg/day when body weight < 50kg or 600 mg/day when body weight was ≥ 50kg, pyrazinamide 1500mg/day and ethambutol 750mg/day for two month. After two-months, pyrazinamide as discontinued and the remaining drugs were used for a total of 6 months. The study was approved by the ethical committee of the West China Hospital, Sichuan University. Written informed consent was obtained from all patients enrolled.

### Patient Monitoring and Diagnosis of ATDH

In the prospective study, liver function tests were performed at 2 weeks, 1 month, 2 months and 3 months of the start of therapy or whenever the participants manifested symptoms of suspected hepatitis such as loss of appetite, nausea, vomiting, fever, and jaundice. In the retrospective study, in addition to abovementioned time point, liver function was also monitored monthly after 3 months up to 6 months. ATDH was diagnosed with International consensus criteria defined as 1) alanine aminotransferase (ALT) level at least greater than two-fold of the upper limit of normal (ULN), and/or a combined increase in aspartate aminotransferase (AST) and total bilirubin provided one of them was greater than two-times of ULN during the treatment; 2) causality assessment result was highly probable (>8), probable (6–8), possible (3–5) based on the the Roussel Uclaf Causality Assessment Method (RUCAM) scale [16, 17].

### Selection of tag-SNPs

All SNPs in the *GSTP1* gene were downloaded from the Chinese Han in Beijing (CHB) database of HapMap (http://www.hapmap.org/, accessed 25 April 2015),in a region 3,000 base pairs upstream and 3,000 base pairs downstream of *GSTP1*. All SNPs were filtered using the following criteria: (i) Hardy–Weinberg equilibrium (HWE) test P ≧ 0.05; (ii) r^2^ of pairwise linkage disequilibrium (LD) ≤ 0.8; and (iii) minor allele frequency (MAF) ≧ 0.05. Then, tag-SNPs were processed and chosen using Haploview 4.2 software (Broad Institute of MIT and Harvard, Cambridge, MA, USA) according to their ability to tag most of the remaining variants after determining LD patterns.

### Sample preparation and genotyping

Following enrollment into the study, a peripheral blood specimen was collected from each patient in ethylenediamine tetraacetic acid (EDTA) coated tubes (BD Vacutainers, Franklin Lakes, NJ, USA), processed with a commercial DNA extraction kit (Axygen Scientific Inc, Union City, CA, USA) and stored at -80°C for further analysis.

The *GSTP1* SNP genotyping for majority of the prospective study samples was based on the Sequenom MassARRAY iPLEX platform that utilizes matrix-assisted laser desorption/ionization time-of-flight (MALDI-TOF) mass spectrometry and PCR. The rest of the prospective study samples and all retrospective study samples were genotyped by PCR and improved multiple ligase detection reaction (iMLDR). We confirmed reproducibility by repeat analysis of a randomly chosen subgroup of 5% of study participants. More detailed information regarding the genotyping is available on request.

### Statistical analysis

HWE was calculated using the Chi-squared test. Genotype/allele frequencies were compared in the ATDH and non-ATDH groups utilizing logistic regression analysis, with sex, age, body mass index (BMI) and smoking history as covariates. Linkage disequilibrium and haplotype analyses within our dataset were estimated using the SHEsis online software platform (http://analysis.bio-x.cn). Odds ratios (ORs) and 95% confidence intervals (CIs) were calculated to determine the associations between the risk factors and ATDH. We calculated the power of our study design using the Power and Sample Size Calculation Software (http://biostat.mc.vanderbilt.edu/PowerSampleSize) [18]. Statistical analyses were performed using SPSS (SPSS Inc., Chicago, IL, USA) 19.0 software. P < 0.05 was considered to be statistically significant.

## Results

### Demographics of the study population

**Fig 1:**
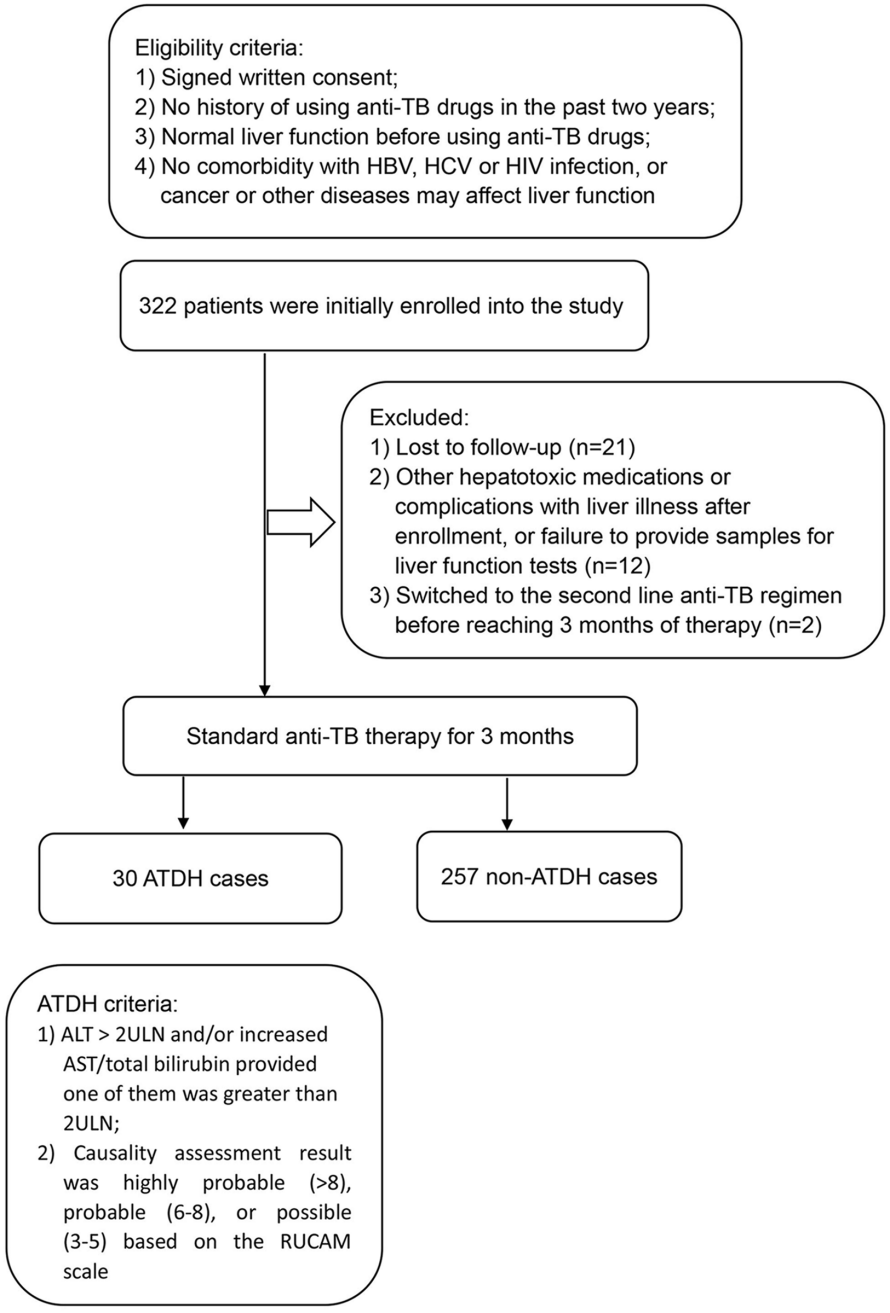
Overall study plan and ATDH outcome stating the inclusion and exclusion criteria in the prospective study.

In the prospective study, 322 patients were initially enrolled to the study, 21 were lost to follow-up, and 12 were excluded due to lack of compliance with the protocol such as use of other hepatotoxic medications or complication with diseases that affect liver function (one case suffered from hepatitis A during follow-up period), or failure to provide samples for liver function tests as scheduled. In addition, 2 patients failed the first line anti-TB therapy and switched to second line anti-TB regimen before reaching 3 months of therapy. The remaining 287 TB patients who had completed 3-months of first line anti-TB therapies and follow up were assessed. Among the 287 patients, 30 (10.5%) had an ALT/AST/total bilirubin levels above two times of the ULN. Fig 1 illustrates the overall study plan and ATDH outcome with the inclusion and exclusion criteria. The baseline characteristics of all participants of the two study samples are summarized in Table 1. There was no statistically significant difference in the distribution of sex, age, weight, BMI and smoking history between the ATDH and non-ATDH groups. Prior to treatment, all participants had normal ALT, AST, and total bilirubin values, and there was no significant difference between the two groups (P > 0.05).

**Table 1:** Characteristics of patients with and without ATDH.

Characteristic	Patients with ATDH	Patients without	*P* value
Prospective study	N = 30	N = 257	
Sex (male/female)	11/19	101/156	0.845
Age (years)^a^	34.3±15.76	38.75±15.76	0.136
Weight (kg)^a^	55.35±8.12	54.64±10.36	0.726
BMI (kg/m^2^)^a^	21.68±2.67	20.65±3.68	0.161
Smoking history	7 (23.3%)	68 (26.5%)	0.828
Retrospective study	N = 115	N = 116	
Sex (male/female)	66/49	69/47	0.790
Age (years)^a^	39.26±17.00	38.17±17.15	0.629
Weight (kg)^a^	55.10±8.55	56.61±9.46	0.203
BMI (kg/m^2^)^a^	20.42±2.59	20.83±3.33	0.318
Smoking history	33 (28.7%)	42 (36.2%)	0.322

^a^ Values are presented as mean ± SD(range)

### *GSTP1* polymorphisms in patients with and without ATDH

Two tag-SNPs (rs1695 and rs4147581) were selected for genotyping. Rs1695 was selected because it was a missense SNP (leading to an amino acid change). It was a surrogate for other 8 SNPs in high LD (r^2^ > 0.8) with it. Rs4147581 was a singleton in the HapMap database of Chinese Han individuals. The distance between the two SNPs is 1104 base-pairs. The associations between SNPs and ATDH were analyzed with logistic regression adjusted for sex, age, BMI and smoking history. The genotype distributions of the two SNPs in the ATDH and non-ATDH groups of the two samples are shown in Table 2. In the initial prospective study, significant differences were found in allele/genotype distributions of rs1695 rs4147581 between ATDH and non-ATDH groups. Compared with rs1695 allele G, allele A was associated with susceptibility to the development of ATDH (OR = 3.876, 95%CI: 1.258–11.91; P = 0.018) and the AA genotype (recessive model) was related to a significantly increased risk of ATDH than the GG+AG genotype (OR = 3.68, 95%CI: 1.18–11.36, P = 0.025). Rs4147581 GG genotype (dominant model) was related to a significantly increased risk of ATDH than the CC+GC genotype (OR = 2.578, 95% CI: 1.076–6.173; P = 0.034).

**Table 2:** *GSTP1* polymorphisms in patients with and without ATDH.

SNPs	ATDH group N (%)	Non-ATDH group N (%)	OR* (95%CI)	P*
Prospective study				
rs1695 (A>G)				
Allele				
G	7 (11.7)	102 (19.9)	Reference	
A	53 (88.3)	410 (80.1)	3.876 (1.258–11.90)	0.018
Genotype				
GG+AG**	6 (20)	93 (36.3)	Reference	
AA	24 (80)	163 (63.7)	3.68 (1.18–11.36)	0.025
rs4147581 (G>C)				
Allele				
C	15 (25)	164 (32.3)	Reference	
G	45 (75)	344 (67.7)	1.938 (0.944–3.984)	0.071
Genotype				
GC+CC**	12 (40)	140 (55.1)	Reference	
GG	18 (60)	114 (44.9)	2.578 (1.076–6.173)	0.034
Retrospective study				
rs1695 (A>G)				
Allele				
G	24(10.5)	44(19.3)	Reference	
A	204(89.5)	184(80.7)	2.10 (1.17–3.76)	0.012
Genotype				
GG+AG**	24(21.1)	39(34.2)	Reference	
AA	90(78.9)	75(65.8)	2.00 (1.05–3.83)	0.035
rs4147581 (G>C)				
Allele				
C	55(24.3)	71(31.1)	Reference	
G	171(75.7)	157(68.9)	1.295 (0.831–2.020)	0.253
Genotype				
GC+CC**	48(42.5)	57(50)	Reference	
GG	65(57.5)	57(50)	1.239 (0.704–2.179)	0.458

OR, Odds Ratio; CI, confidence interval

* Adjusted for sex, age, BMI and smoking history with logistic regression.

** Due to the small number of minor allele homozygotes, this genotype was combined with heterozygotes in the analysis.

The D’ and r^2^ between rs4147581 and rs1695 were 1.000 and 0.504, respectively in this study. In order to test if the association of rs4147581 with ATDH was dependent on rs1695, the association was adjusted in the regression model for rs1695, in addition to sex, age, BMI and smoking history. The results showed that rs4147581 was not independently associated with ATDH. Therefore, the association of rs4147581 with ATDH was likely due to its LD with rs1695.

To verify the finding of the prospective study, we analyzed an independent retrospective study group. The distribution of alleles/genotypes at rs1695 was also significantly associated with ATDH. Compared with the G allele, allele A was associated with a significant increased risk of ATDH (OR = 2.10, 95%CI: 1.17–3.76; p = 0.012). We also found that rs1695 genotype was related to ATDH. Compared with GG+AG, the AA genotype was associated with a significantly increased risk of ATDH (OR = 2.00, 95%CI: 1.05–3.83; p = 0.035). No significant relationship was detected between rs4147581 allele/genotype distributions and ATDH risk.

### Haplotype analysis

In the haplotype analysis, haplotypes with frequency < 0.03 were ignored, and in the prospective study, we did not detect a significant difference in haplotype distribution between the two groups. However, in the retrospective study we found that the GC haplotype was significantly associated with a decreased risk of ATDH (Table 3).

**Table 3:** Haplotype analysis of rs1695 and rs4147581.

Haplotype	ATDH group N (%)	Non-ATDH group N (%)	OR* (95%CI)	P*
Prospective study				
A C	8 (13.3)	64 (12.6)	1.067 (0.485–2.350)	0.871
A G	45 (75.0)	344 (67.7)	1.430 (0.775–2.641)	0.251
G C	7 (11.7)	100 (19.7)	0.539 (0.238–1.221)	0.133
Retrospective study				
A C	31 (13.7)	27 (11.8)	1.183 (0.681–2.056)	0.550
A G	171 (75.7)	157 (68.9)	1.406 (0.93–2.126)	0.105
G C	24 (10.6)	44 (19.3)	0.497 (0.291–0.849)	0.010

* Adjusted for sex, age, BMI and smoking history with logistic regression.

### Power analysis

In order to assess the power of our study design we used relative risks (RR) of 2.0, 3.0, and 4.0 to calculate the power of the sample size for the two SNPs under an allelic model. The results showed that our study has reasonable power (>80%) to draw conclusions with RR 3.0 or above (Table 4).

**Table 4:** Power of the study with different relative risks (RR).

SNP	Genetic model	MAF of control	Power %		
			RR = 2	RR = 3	RR = 4
Prospective cohort					
rs1695	Allele	0.199	0.65	0.96	0.99
rs4147581	Allele	0.323	0.72	0.98	0.99
Retrospective cohort					
rs1695	Allele	0.193	0.99	1	1
rs4147581	Allele	0.311	0.99	1	1

SNP, single nucleotide polymorphism; RR, relative risk

## Discussion

The safety of anti-TB drug therapy shows large inter-individual variation, even in patients treated with the same drugs and a standard dosing regimen. Demographic and clinical factors contribute to this variability, genetic factors play important roles as well [19]. A significant number of studies have evaluated the roles of *GST* polymorphisms on the incidence of ATDH. The majority of these studies were conducted to investigate the association of *GSTM1* and/or *GSTT1* polymorphisms with ATDH [20], The *GSTM1* null genotype was significantly associated with increased ATDH risk [21]. However, studies of *GSTP1* with ATDH have been rare. *GSTP1*, encoding the π-class of enzymes which accounts for approximately 90% of the enzymatic activity of the GST family [22], may be more important in the development of ATDH.

In the initial prospective study, we assessed the association of two tag-SNPs of *GSTP1* and the development of ATDH in a Chinese TB population. Significant associations were found for both rs1695 and rs4147581 with ATDH. In order to verify this result, we conducted an independent retrospective study which revealed that rs1695 A allele and AA genotype were significantly associated with ATDH. The results of the retrospective study supported the finding of our prospective study. This is the first study to provide evidence that SNPs in *GSTP1* are related to ATDH.

Our results have shown that subjects with rs1695A allele were susceptible to ATDH. Several lines of evidence also suggested that these results were true positives: 1) rs1695 (A>G) is a functional SNP, which leads to the Ile105Val amino acid substitution; it has been shown to result in altered catalytic activity [23, 24]. Subjects homozygous for the rs1695A allele (105Ile) had decreased *GSTP1* mRNA expression compared to those with at least one rs1695G allele (105Val) [25] and had lower antioxidant activities. This provides a rationale for subjects with the1695A allele being susceptible to ATDH. 2) The study of He *et al*. has shown that hypermethylation of the *GSTP1* promoter was associated with ATDH [15]. Subjects with hypermethylation of the *GSTP1* promoter showed lower expression of *GSTP1*, and were susceptible to ATDH. 3) Ts1695 has been associated with other liver diseases. For example, the homozygous rs1695A genotype was reported to be associated with an 8-fold increase in the risk of liver disease compared with other *GSTP1* genotypes (P = 0.002) in pediatric patients with cystic fibrosis [26], which was similar to our results that rs1695A was associated with liver injury of ATDH. In addition, hepatocellular carcinoma patients carrying the *GSTP1* Val/Val (rs1695GG) genotype had significantly better survival than those carrying the *GSTP1* IIe/IIe (rs1695AA) genotype [27]. 4) *GSTP1* rs1695 was found to be related to therapeutic response and occurrence of adverse drug effect in chemotherapy of cancers such as gastric cancer [28], colorectal cancer [29], nonsmall cell lung cancer [30], ovarian cancer [31], esophageal cancer [32], and breast cancer [33]. 5) Most importantly, we replicated our result with independent samples, which significantly increased the reliability of the results.

To date, only one study has investigated the relationship between *GSTP1* SNPs and ATDH and no association was found [15]. The inconsistent results between our data and those of He *et al*. could be attributed to several factors. Firstly, the study populations might be different in the two studies. Our subjects were Chinese Han subjects, while subjects in the He *et al*. study might not be from the Chinese Han population. The MAF of rs1695(A>G) in non-ATDH group in our two samples were 19.9% and 19.3%, respectively, which was similar to the MAF of the Chinese Han population in the HapMap database (18.38%). While in He *et al*. study, the MAF of rs1695 in control subjects was 30.71% [15]. Secondly, the study designs were different. Our first sample was a prospective cohort study, which meant that it was less likely to be subjected to recall bias, as well as other possible biases caused by self-selection of patients, while the study of He *et al*. was a cross-sectional study. Thirdly, as discussed above, both *GSTP1* rs1695 and methylation of the *GST* promoter were associated with expression of the *GSTP1* gene, therefore, could be associated with ATDH. Since rs1695 was not associated with promoter hypermethylation status [34], we reasoned that rs1695 and promoter methylation of *GSTP1* contribute to ATDH independently.

Our study also showed that the rs4147581GG genotype was associated with a significantly increased risk of ATDH in a dominant model. However, we did not detect this significance in the retrospective study. Rs4147581 was a singleton in the HapMap database. The D’ and r^2^ between rs4147581 and rs1695 were 1.000 and 0.504, respectively in our study. In order to address the possibility that the association between rs4147581 and ATDH was due to LD between rs1695 and rs4147581, regression analysis including both SNPs was performed and the results suggested that the associations of ATDH with rs4147581 were due to its LD with rs1695. Only one study reported that rs4147581 was significantly correlated with overall survival in hepatocellular carcinoma (HCC) patients in the literature [35].

In conclusion, we evaluated the impacts of *GSTP1* tag-SNPs on the development of ATDH in two independent samples. The *GSTP1* tag-SNPs influenced the risk of ATDH in a Chinese Han population. More studies with larger sample size and with different ethnic populations are warranted to verify our findings. Investigations of genetic susceptibility to ATDH have shed a light on TB personalized therapy [36]. Therefore, studies of genetic factors in ATDH have great potential in guiding anti-TB treatment.
